# Early surrogates of functional outcome after thrombectomy for MCA-M2 occlusions

**DOI:** 10.1038/s41598-025-34777-8

**Published:** 2026-02-17

**Authors:** Helge C. Kniep, Lukas Meyer, Gabriel Broocks, Matthias Bechstein, Christian Heitkamp, Laurens Winkelmeier, Tobias Faizy, Ludger Feyen, Caspar Brekenfeld, Fabian Flottmann, Maximilian Schell, Uta Hanning, Götz Thomalla, Jens Fiehler, Susanne Gellißen, the German Stroke Registry-Endovascular Treatment (GSR-ET), Arno Reich, Omid Nikoubashman, Christian Nolte, Eberhard Siebert, Alexander Nave, Charlotte Pietrock, Gabor Petzold, Franziska Dorn, Jan Hendrik Schäfer, Fee Keil, Anna Alegiani, Joachim Röther, Bernd Eckert, Maximilian Schell, Götz Thomalla, Fabian Flottmann, Jens Fiehler, Mario Abruscato, Sven Thonke, Timo Uphaus, Klaus Gröschel, Peter Schellinger, Jan Borggrefe, Lars Kellert, Hanna Zimmermann, Steffen Tiedt, Silke Wunderlich, Tobias Boeckh-Behrens, Annerose Mengel, Ulrike Ernemann

**Affiliations:** 1 Department of Diagnostic and Interventional Neuroradiology, University Medical Center Hamburg-Eppendorf, Martinistraße 52, 20246 Hamburg, Germany; 2 Department of Neurology, University Medical Center Hamburg-Eppendorf, Hamburg, Germany; 3 Department of Diagnostic and Interventional Radiology, Helios Klinikum Krefeld, Krefeld, Germany; 4 Faculty of Health, School of Medicine, University Witten/Herdecke, Witten, Germany; 5 Department of Diagnostic and Interventional Radiology, HELIOS University Hospital Wuppertal, University Witten/Herdecke, Wuppertal, Germany; 6 Department of Neuroradiology, HELIOS Medical Center, Campus of MSH Medical School Hamburg, Schwerin, Germany; 7 Department of Neuroradiology, University Medical Center Mannheim, Mannheim, Germany; 8 Department of Radiology, University Medical Center Münster, Münster, Germany; 9 Department of Neurology, University Hospital, RWTH Aachen University, Aachen, Germany; 10 Department of Neuroradiology, University Hospital, RWTH Aachen University, Aachen, Germany; 11 Department of Neurology, Charité – Universitätsmedizin Berlin corporate member of Freie Universität Berlin and Humboldt-Universität zu Berlin, Berlin, Germany; 12 Department of Neuroradiology, Charité – Universitätsmedizin Berlin corporate member of Freie Universität Berlin and Humboldt-Universität zu Berlin, Berlin, Germany; 13 Department of Neurology, University Hospital Bonn, Bonn, Germany; 14 Department of Neuroradiology, University Hospital Bonn, Bonn, Germany; 15 Department of Neurology, University Hospital Frankfurt, Goethe University, Frankfurt am Main, Germany; 16 Department of Neuroradiology, University Hospital Frankfurt, Goethe University, Frankfurt am Main, Germany; 17 Department of Neurology, Krankenhaus Reinbek St. Adolf-Stift, Reinbek, Germany; 18 Department of Neurology, Asklepios Klinik Hamburg Altona, Hamburg, Germany; 19 Department of Neuroradiology, Asklepios Klinik Hamburg Altona, Hamburg, Germany; 20 Department of Neurology, University Medical Center Hamburg-Eppendorf, Hamburg, Germany; 21 Department of Neuroradiology, University Medical Center Hamburg-Eppendorf, Hamburg, Germany; 22 Department of Neurology, Klinikum Hanau, Hanau, Germany; 23 Department of Neurology, University Medical Center of the Johannes Gutenberg University Mainz, Focus Program Translational Neurosciences (FTN), Mainz, Germany; 24 Department of Neurology, University Medical Center of the Johannes Gutenberg University Mainz, Mainz, Germany; 25 Department of Neurology, Mühlenkreiskliniken, Johannes Wesling Klinikum Minden, Minden, Germany; 26 Department of Radiology, Mühlenkreiskliniken, Johannes Wesling Klinikum Minden, Minden, Germany; 27 Department of Neurology, Ludwig Maximilian University (LMU), Munich, Germany; 28 Department of Neuroradiology, Ludwig Maximilian University (LMU), Munich, Germany; 29 Institute for Stroke and Dementia Research, Ludwig Maximilian University (LMU), Munich, Germany; 30 Department of Neurology, Klinikum rechts der Isar, School of Medicine, Technical University of Munich, Munich, Germany; 31 Department of Neuroradiology, Klinikum rechts der Isar, School of Medicine, Technical University of Munich, Munich, Germany; 32 Department of Neurology & Stroke, University Hospital Tübingen; Hertie Institute for Clinical Brain Research, University of Tübingen, Tübingen, Germany; 33 Department of Neuroradiology, University of Tübingen, Tübingen, Germany

**Keywords:** Stroke, Distal occlusion, M2 occlusion, Outcome, Prognosis, Early neurological improvement, Thrombectomy, Neurology, Stroke

## Abstract

The National Institutes of Health Stroke Scale (NIHSS) at 24h is a strong predictor of long-term independence after mechanical thrombectomy for anterior circulation strokes. Its predictive value in patients with M2 segment of the middle cerebral artery (MCA-M2) occlusions, however, remains unclear. This study compares NIHSS scores at admission and 24h and early neurological improvement (ENI) definitions to predict functional outcomes at 90d for M2 occlusions. Patients with isolated MCA-M2 occlusions from the German Stroke Registry (06/2015–12/2021) were analyzed. NIHSS at admission, 24h and ENI definitions were compared for predicting excellent and good outcome, defined as modified Rankin Scale (mRS) 0-1 and 0-2. Factors impairing predictive power were identified based on multivariable logistic regression. Among 1,268 patients, 24h NIHSS showed the highest predictive accuracy, with an area under the curve (AUC) of 0.85 [95% Confidence Interval: 0.83-0.87] for both good and excellent outcomes. Optimal NIHSS thresholds were ≤8 for good and ≤7 for excellent outcomes. Advanced age, higher pre-stroke mRS, diabetes, and higher procedure passes significantly reduced prognostic accuracy. NIHSS at 24h, with thresholds of ≤8 and ≤7, is the best surrogate for predicting good and excellent functional outcomes in MCA-M2 occlusion patients. Prognostic value diminishes with advanced age, pre-stroke disability, and comorbidities such as diabetes.

**Supplementary Information:**

The online version contains supplementary material available at 10.1038/s41598-025-34777-8.

## Introduction

In recent years, numerous randomized trials have unequivocally demonstrated the safety and efficacy of mechanical thrombectomy (MT) in addressing large vessel occlusion (LVO) strokes, which are defined as occlusions in the intracranial internal carotid artery (ICA) and the M1 segment of the middle cerebral artery (MCA)^1^. For patients with LVO, MT has therefore been established as standard of care in international guidelines^1,2^. However, 24% to 40% of acute ischemic strokes are caused by medium and distal occlusions^3^. Emerging data suggest that MT might also be safe and effective for medium and distal occlusions^4–6^. Furthermore, MT has been recently emphasized by an international consensus as an encouraging option for medium and distal occlusions^3^ and is now increasingly performed for these occlusion sites^7^. While emerging data from observational studies and meta-analyses suggest that MT may also be safe and effective in medium and distal occlusions^4,5,8–11^, three randomized controlled trials (DISTAL^12^, ESCAPE-MeVO^13^, DISCOUNT^14^) did not report results favoring MT plus best medical treatment vs. BMT alone. In DISTAL and ESCAPE-MeVO, thrombectomy plus best medical management did not improve functional outcomes at 90 days compared with best medical management alone, while DISCOUNT was terminated early for futility and safety concerns. Improved insight into the factors influencing functional outcome after MT for medium and distal occlusions could facilitate more precise identification of patients who are likely to derive benefit from the intervention.

Early prediction of long term functional outcomes in patients with LVO strokes has been investigated in previous works. Among various definitions of early neurological improvement that have been reported in literature, the National Institute of Health Stroke Scale (NIHSS) score at 24 h was lately found to be the most accurate surrogate marker for prediction of functional outcome at day 90 after thrombectomy for anterior and posterior circulation stroke (ACS, PCS)^15–18^. In particular, the cut-off at 8 NIHSS points was found to be independently associated with good functional outcome defined as modified Rankin Scale (mRS) 0 - 2 at day 90 in patients with ACS^15,19^.

However, it has been emphasized that the NIHSS and mRS scales might perform differently in patients with occlusions of the M2 segment of the middle cerebral artery (MCA-M2). NIHSS and mRS scales are limited in their granularity and heavily focused on motor function and thus unable to reflect domain-specific impairments (i.e. eloquence) that often play a dominant role in distal and medium vessel occlusion (DMVO) stroke-related disability^20^. Furthermore, based on a smaller area that is affected by ischemia, clinical outcomes can be expected to be better than in LVO stroke patients. While there are some data on predictors of a good clinical outcome based on smaller sample sizes, the effect of early neurological improvement (ENI) remains unstudied in this population^21–24^. Also, reliability of the NIHSS at 24 h as early surrogate marker for long-term outcome and the impact of specific possible factors reducing the prognostic value have not been analyzed in detail for DMVO.

This study investigated the value of established ENI definitions and NIHSS scores at admission and at 24 h as surrogate marker for long-term functional outcome after thrombectomy specifically in patients with MCA-M2 occlusion. Furthermore, procedure-related factors and individual patient characteristics reducing the prognostic value are identified.

We hypothesized that (1) the NIHSS at 24 h serves best as reliable surrogate of long-term functional independency after MCA-M2 thrombectomy and (2) procedure-related and individual patient characteristics impact the prognostic value of surrogate markers.

## Methods

### Study design and participating centers

The German Stroke Registry-Endovascular Treatment (GSR-ET) is an ongoing, open-label, prospective, multicenter registry of 25 sites in Germany collecting consecutive patients undergoing thrombectomy (ClinicalTrials.gov Identifier: NCT03356392). The study was approved by the ethics committee of the chamber of physicians at Ludwig-Maximillians University (LMU), Munich (689-15) as the leading ethics committee, approval by local ethics committees or institutional review boards was obtained for all participating sites according to local regulations. The study was conducted in accordance with the Declaration of Helsinki^25^. Patients, if capable, or their legal representatives, if available, were asked to provide written informed consent. As all information was collected within clinical routine, patients could be enrolled and information on baseline and in-hospital treatment recorded even if patients were incapable of providing consent and no legal representative was available. Informed consent was obtained at the latest prior to the follow-up assessment 90 days after stroke. A detailed description of the GSR-ET study design and the major findings have been published recently^26,27^

### Study cohort

All patients with MCA-M2 occlusion enrolled in the GSR-ET (June 2015–December 2021) were analyzed. The main inclusion criteria of GSR-ET are diagnosis of acute ischemic stroke, initiation of an endovascular procedure for treatment, and age ≥18 years, according to national guidelines. There are no exclusion criteria. For this analysis, all patients that met the following criteria were included: a) isolated primary MCA-M2 occlusion; and b) availability of neurological status at baseline, 24 h and 90 days, recanalization success measured in the modified Thrombolysis in Cerebral Infarction (mTICI) scale^28^ and relevant clinical baseline parameters (complete case analysis). The analysis was conducted in accordance with the Strengthening the reporting of observational studies in epidemiology (STROBE) guidelines.

### Clinical and radiologic assessment

Patient characteristics, radiologic findings and treatment outcomes were obtained from the GSR-ET. Clinical assessments and reading of baseline imaging, digital subtraction angiograms and follow-up imaging were conducted by local investigators at each participating center (single reader). For occlusions of the middle cerebral artery (MCA), the GSR-ET registry differentiates proximal M1, distal M1 and MCA-M2 occlusions, evaluation of arterial dominance is not part of the registry protocol. Assessment of the occlusion location was performed by the treating neurointerventionalist. Clinical assessments were performed at baseline and at 90 days using the NIHSS and the mRS. Reperfusion success was evaluated by the treating neurointerventionalist based on intraprocedural DSA images using mTICI scale.

### Outcomes and safety events

Primary endpoints were excellent functional outcome defined as mRS 0–1 at day 90 after the index procedure and good functional outcome defined as mRS 0–2 at day 90 after the index procedure. Established predictors of long-term functional outcome in acute ischemic stroke were evaluated for their ability to predict good functional outcome in patients with MCA-M2 occlusion. Two binary definitions of early neurological improvement were applied according to previous studies based on the NIHSS at 24h: a) NIHSS improvement ≥8 points from baseline or reaching ≤1 points at 24 h (major early neurological improvement [mENI])^29,30^, b) NIHSS improvement ≥10 points from baseline or reaching 0 points at 24 h (dramatic early neurological improvement [dENI])^31,32^. In addition, NIHSS change (ΔNIHSS: NIHSS 24 h − NIHSS Admission) and NIHSS percentage change (% NIHSS: [NIHSS 24 h − NIHSS Admission]/0.5 [NIHSS 24 h + NIHSS Admission]) were assessed for their ability to predict functional outcome in patients with MCA-M2 occlusion. NIHSS percentage change was calculated using RPD (relative percentage difference) to minimize the number of cases with denominator 0.

Safety events were adverse events during treatment defined according to the GSR-ET registry data base, specifically device malfunctions, vasospasms, clot migration and embolization, dissections and perforations, intraprocedural strokes and intracranial hemorrhage (ICH).

### Statistical analysis

Standard descriptive statistics were used for all study end points. Univariable distribution of metric variables was described with mean and interquartile range (IQR) and categorical variables with absolute and relative frequencies.

Receiver operating characteristics area under the curve (ROC AUC) analysis with maximization of the Youden index (sensitivity + specificity − 1) was conducted to derive optimal cut-off thresholds^33^. Prognostic stability of the optimal metric was evaluated for subgroups stratified for age (≤65 years; 66–80 years; >80 years) and severity of symptoms at admission (mild: NIHSS <10; moderate: 10–15; severe: >15).

Multivariable logistic regression with Akaike information criterion (AIC)-based stepwise backward variable selection was performed to identify factors that increase probability of reaching the optimal surrogate threshold at 24 h for good and excellent long-term functional outcome. Additional subgroup analyses were performed to identify factors leading to good and excellent functional outcome at day 90 in patients that did not reach the relevant thresholds at 24 h and vice versa. Odds ratio (OR) with 95% confidence intervals (CIs) and p values were calculated for selected variables. A two-sided P value <0.05 was considered to be statistically significant. All analysis were performed using R 4.3.1.

## Results

### Baseline, procedural and outcome characteristics

**Fig. 1:**
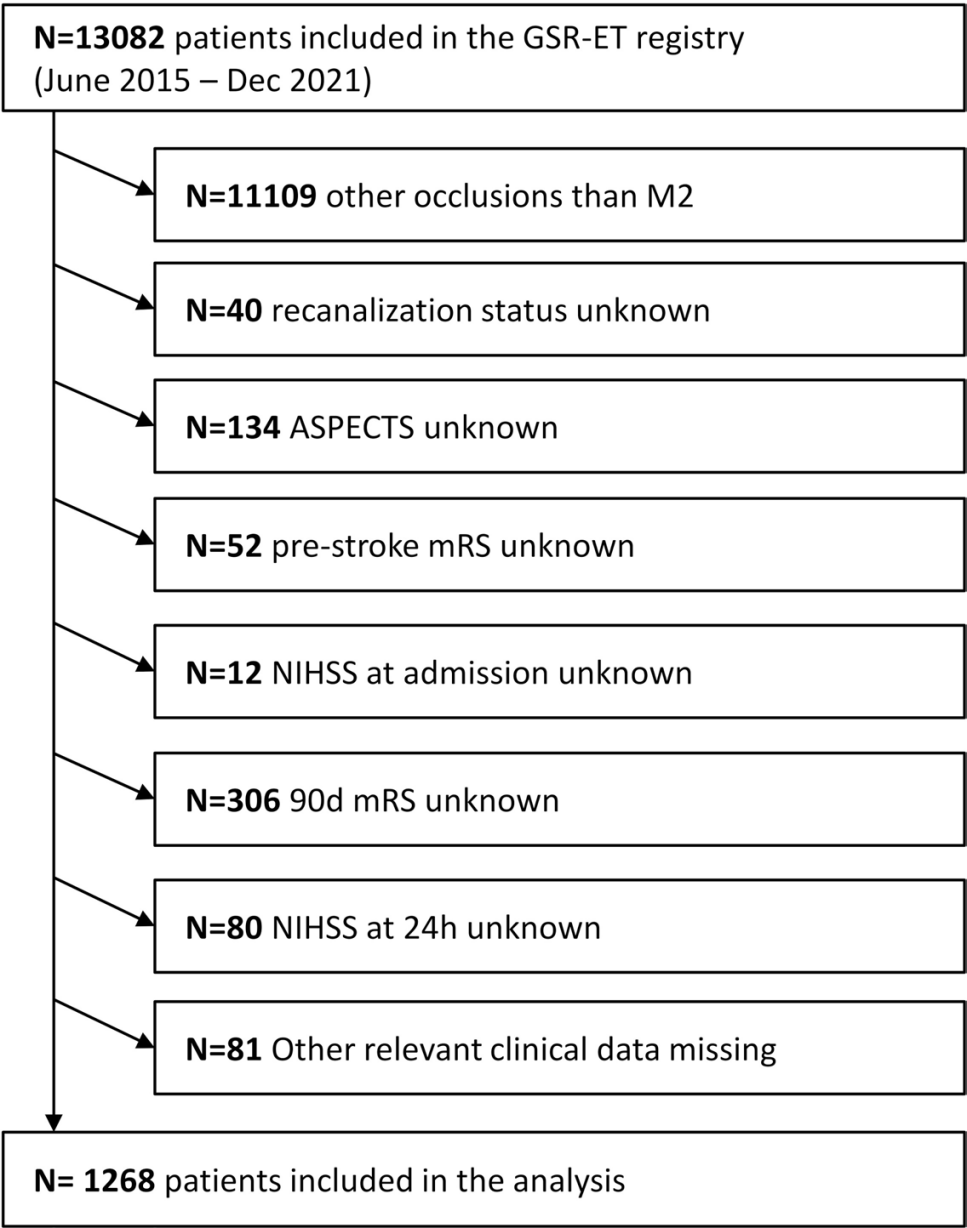
Patient inclusion flow chart.

In total, 1268 patients fulfilled the inclusion criteria and required data points were available (Fig. 1). To assess potential selection bias, we compared baseline characteristics and clinical outcomes between included and excluded patients; no significant differences were observed, supporting the robustness of the complete-case analysis (Supplementary Table S1). Overall, 1064 (83.9%) patients were recanalized successfully and 387 (30.5%) patients achieved excellent functional outcome with mRS ≤ 1 at day 90, while 566 (44.6 %) patients received good functional outcome with mRS ≤2 at day 90.

Patients with good functional outcome and without good functional outcome were significantly different in age (mean 69.8 vs. 78.0 years), sex (male) (53.2% vs. 45.0%), pre-stroke mRS (mean 0.1 vs. 1.2), NIHSS at admission (mean 8.5 vs. 12.6), prevalence of hypertension (71.9% vs. 83.5%), prevalence of diabetes (17% vs. 27.5%), prevalence of atrial fibrillation (36.9% vs. 51.4%), ASPECTS (mean 9 vs. 8.7), administration of intravenous thrombolysis (IVT) (57.2% vs. 40.6%), number of passes (mean 1.7 vs. 2.2), share of successful recanalization (93% vs. 77%), any adverse event during treatment (15.7% vs. 23.9%) and NIHSS at 24 h (mean 4.8 vs. 14.3) (Table 1).

**Table 1:** Studie cohort clinical characteristics.

Variable	mRS>2 (N=702)	mRS≤2 (N=566)	p-value	mRS>1 (N=881)	mRS≤1 (N=387)	p-value	Total (N=1268)
Age			< 0.001			< 0.001	
- Mean (SD)	78.0 (10.5)	69.8 (13.6)		76.8 (11.5)	68.8 (13.5)		74.3 (12.7)
- Q1, Q3	72.2, 85.0	61.0, 80.0		71.0, 85.0	60.0, 79.0		68.0, 83.0
Sex female	386 (55.0%)	265 (46.8%)	0.004	478 (54.3%)	173 (44.7%)	0.002	651 (51.3%)
Pre-stroke mRS			< 0.001			< 0.001	
- Mean (SD)	1.2 (1.4)	0.1 (0.4)		1.0 (1.3)	0.1 (0.2)		0.7 (1.2)
- Q1, Q3	0.0, 2.0	0.0, 0.0		0.0, 2.0	0.0, 0.0		0.0, 1.0
NIHSS admission			< 0.001			< 0.001	
- Mean (SD)	12.6 (6.1)	8.5 (5.3)		12.0 (6.1)	7.9 (5.0)		10.7 (6.1)
- Q1, Q3	8.0, 17.0	5.0, 11.8		7.0, 16.0	4.0, 11.0		6.0, 15.0
Hypertension	586 (83.5%)	407 (71.9%)	< 0.001	719 (81.6%)	274 (70.8%)	< 0.001	993 (78.3%)
Diabetes	193 (27.5%)	96 (17.0%)	< 0.001	228 (25.9%)	61 (15.8%)	< 0.001	289 (22.8%)
Dyslipidemia	338 (48.1%)	256 (45.2%)	0.301	408 (46.3%)	186 (48.1%)	0.565	594 (46.8%)
Atrial fibrillation	361 (51.4%)	209 (36.9%)	< 0.001	443 (50.3%)	127 (32.8%)	< 0.001	570 (45.0%)
ASPECTS			< 0.001			< 0.001	
- Mean (SD)	8.7 (1.5)	9.0 (1.3)		8.7 (1.5)	9.1 (1.3)		8.8 (1.4)
- Q1, Q3	8.0, 10.0	8.0, 10.0		8.0, 10.0	8.0, 10.0		8.0, 10.0
Occlusion side left	409 (58.3%)	329 (58.1%)	0.961	519 (58.9%)	219 (56.6%)	0.440	738 (58.2%)
I.v. thrombolysis	285 (40.6%)	324 (57.2%)	< 0.001	373 (42.3%)	236 (61.0%)	< 0.001	609 (48.0%)
# of passes			< 0.001			< 0.001	
- Mean (SD)	2.2 (1.7)	1.7 (1.2)		2.1 (1.6)	1.7 (1.2)		2.0 (1.5)
- Q1, Q3	1.0, 3.0	1.0, 2.0		1.0, 3.0	1.0, 2.0		1.0, 3.0
mTICI 2b-3	540 (76.9%)	524 (92.6%)	< 0.001	707 (80.2%)	357 (92.2%)	< 0.001	1064 (83.9%)
Treatment AE	168 (23.9%)	89 (15.7%)	< 0.001	197 (22.4%)	60 (15.5%)	0.005	257 (20.3%)
NIHSS at 24h			< 0.001			< 0.001	
- Mean (SD)	14.3 (9.4)	4.8 (5.5)		12.9 (9.4)	3.6 (3.8)		10.0 (9.2)
- Q1, Q3	7.0, 18.0	2.0, 6.0		6.0, 17.0	1.0, 5.0		3.0, 15.0

*NIHSS* National Institutes of Health Stroke Scale, *mRS* modified Rankin Scale, *SD* Standard deviation, Q1, Q2, Q3, first, second (median), third quartile, *ASPECTS* Alberta Stroke Program Early CT Score, *mTICI* modified Thrombolysis in Cerebral Infarction, *AE* Adverse event.

Patients with excellent functional outcome were significantly different from patients that did not achieve excellent outcome after 90 days in age (mean 68.8 vs. 76.8 years)), sex (male) (55.3% vs. 45.7%), pre-stroke mRS (mean 0.1 vs. 1.0) and NIHSS at admission (mean 7.9 vs. 12.0), prevalence of hypertension (70.8% vs. 81.6%) prevalence of diabetes (15.8% vs. 25.9%), prevalence of atrial fibrillation (32.8% vs. 50.3%), ASPECTS (mean 9.1 vs. 8.7), administration of intravenous thrombolysis (IVT) (61.0% vs. 42.3%), number of passes (mean 1.7 vs. 2.1), share of successful recanalization (92.2% vs. 80.2%), any adverse event during treatment (15.5% vs. 22.4%) and NIHSS at 24 h (mean 3.6 vs. 12.9) (Table 1).

### Analysis of early clinical surrogates

**Fig. 2:**
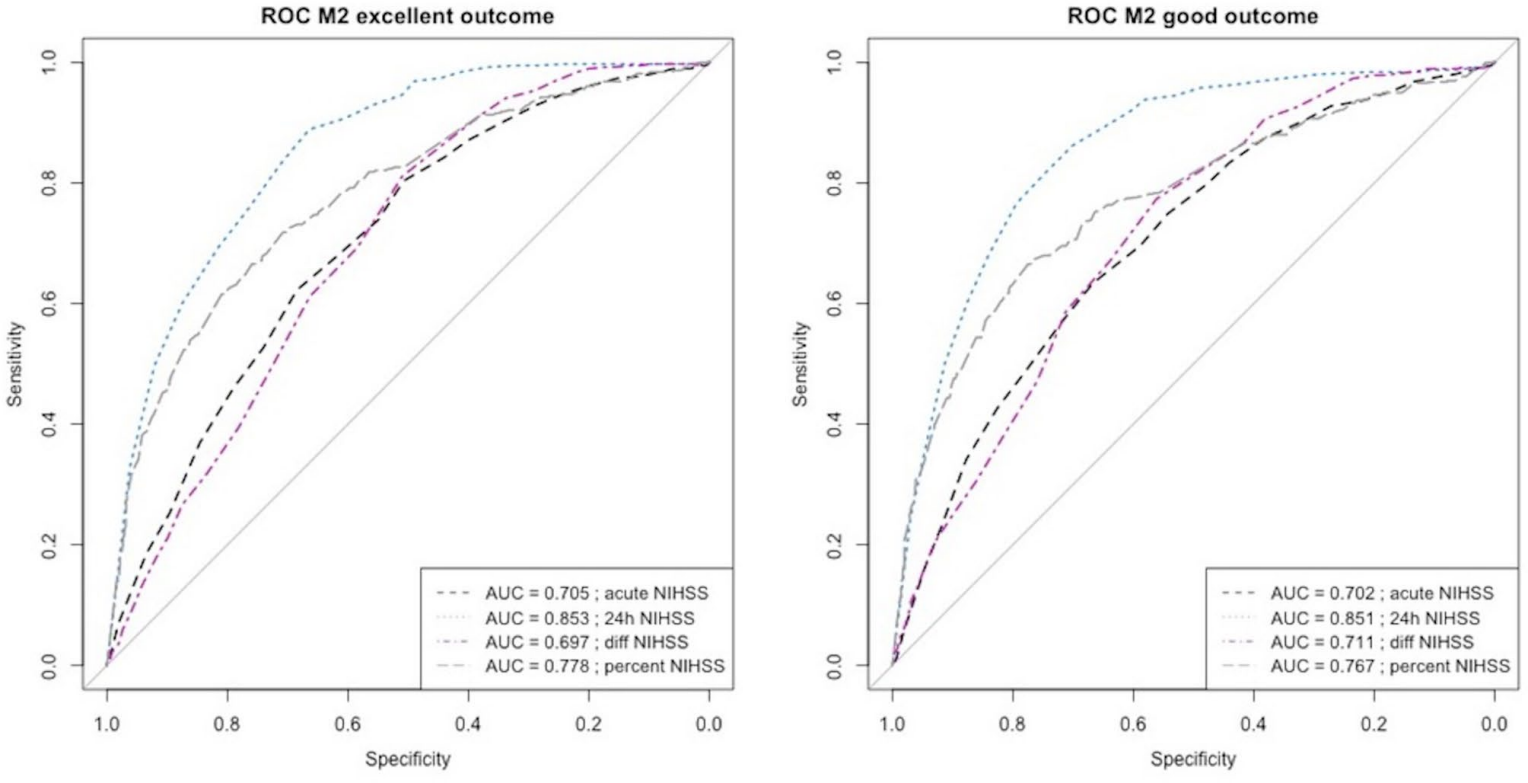
Receiver operating characteristics for prediction of good functional outcome (90d mRS 0–2) and excellent functional outcome (90d mRS 0–1) based on NIHSS at admission, NIHSS at 24 h, NIHSS absolute difference (admission vs. 24 h) and NIHSS percentage change (admission vs. 24 h).

Discriminatory power of the early neurological status and its derived metrics to predict good and excellent functional outcome at 90 days were assessed and are displayed in Table 2 and Fig. 2.

**Table 2:** Discriminatory power and optimal thresholds for predicting good functional outcome (modified Rankin Scale [mRS] ≤2 at 90 days) and excellent functional outcome (mRS ≤1 at 90 days).

Outcome surrogate	Optimal threshold	Sens [95%CI]	Spec [95%CI]	*LR+;* *LR-*	PPV [95%CI]	NPV [95%CI]	Youden index [95%CI]	Accuracy [95%CI]	ROC AUC [95% CI]
90 d mRS ≤2									
dENI	-	0.228 [0.194–0.365.194.365]	0.946 [0.926–0.961.926.961]	4.222; 0.816	0.772 [0.701–0.834.701.834]	0.603 [0.573–0.632.573.632]	0.174 [0.135–0.212.135.212]	0.625 [0.598–0.652.598.652]	-
mENI	-	0.390 [0.350–0.432.350.432]	0.903 [0.879–0.924.879.924]	4.021; 0.676	0.765 [0.711–0.812.711.812]	0.648 [0.617–0.678.617.678]	0.294 [0.248–0.339.248.339]	0.674 [0.648–0.700.648.700]	-
NIHSS admission	9	0.634 [0.593–0.674.593.674]	0.667 [0.630–0.701.630.701]	1.904; 0.549	0.605 [0.565–0.645.565.645]	0.693 [0.657–0.728.657.728]	0.301 [0.248–0.354.248.354]	0.652 [0.625–0.678.625.678]	0.702 [0.675–0.730.675.730]
NIHSS 24h	8	0.862 [0.831–0.890.831.890]	0.701 [0.665–0.735.665.735]	2.883; 0.197	0.699 [0.664–0.733.664.733]	0.863 [0.832–0.890.832.890]	0.563 [0.519–0.607.519.607]	0.773 [0.749–0.796.749.796]	0.851 [0.829–0.873.829.873]
Delta NIHSS	1	0.774 [0.737–0.808.737.808]	0.561 [0.524–0.598.524.598]	1.763; 0.403	0.587 [0551-0.623.623.623.623]	0.755 [0.716–0.791.716.791]	0.335 [0.285–0.385.285.385]	0.656 [0.629–0.682.629.682]	0.711 [0.681–0.738.681.738]
%NIHSS	−30%	0.674 [0.634–0.713.634.713]	0.766 [0.733–797.733]	2.880; 0.426	0.698 [0.657–0.737.657.737]	0.746 [0.712–0.777.712.777]	0.441 [0.391–0.491.391.491]	0.725 []0.700–0.750.700.750]	0.767 [0.741–0.793.741.793]
90 d mRS ≤1									
dENI	-	0.261 [0.218–0.308.218.308]	0.925 [0.906–0.942.906.942]	3.480; 0.799	0.605 [0.526–0.679.526.679]	0.740 [0.713–0.766.713.766]	0.186 [0.139–0.233.139.233]	0.722 [0.697–0.747.697.747]	-
mENI	-	0.455 [0.404–0.506.404.506]	0.872 [0.848–0.893.848.893]	3.555; 0.625	0.609 [0.550–0.666.550.666]	0.784 [0.757–0.810.757.810]	0.327 [0.272–0.381.272.381]	0.744 [0.720–0.768.720.768]	-
NIHSS admission	11	0.804 [0.760–0.842.760.842]	0.507 [0.474–0.541.474.541]	1.631; 0.387	0.417 [0.382–0.454.382.454]	0.855 [0.822–0.884.822.884]	0.311 [0.259–0.363.259.363]	0.598 [0.570–0.623.570.623]	0.705 [0.673–0.734.673.734]
NIHSS 24h	7	0.889 [0.853–0.918.853.918]	0.664 [0.632–0.695.632.695]	2.646; 0.167	0.538 [0.498–0.577.498.577]	0.932 [0.909–0.950.909.950]	0.553 [0.509–0.597.509.597]	0.733 [0.707–0.757.707.757]	0.853 [0.831–0.876.831.876]
Delta NIHSS	1	0.811 [0.769–0.849.769.849]	0.510 [0.476–0.543.476.543]	1.655; 0.371	0.421 [0.385–0.457.385.457]	0.860 [0.827–0.889.827.889]	0.321 [0.270–0.372.270.372]	0.602 [0.574–0.629.574.629]	0.697 [0.670–0.727.670.727]
%NIHSS	−33%	0.718 [0.670–0.763.670.763]	0.708 [0.676–0.738.676.738]	2.459; 0.398	0.516 [0.473–0.559.473.559]	0.853 [0.825–0.878.825.878]	0.426 [0.372–0.481.372.481]	0.711 [0.685–0.736.685.736]	0.778 [0.749–0.807.749.807]

*NIHSS* National Institutes of Health Stroke Scale, *PPV* Positive predictive value, *NPV* Negative predictive value, *LR+* Positive likelihood ratio, *LR*− Negative likelihood ratio, *mENI* major early neurological improvement, *dENI* dramatic early neurological improvement.

### Good outcome ENI definitions

mENI (NIHSS decrease of ≥8 or NIHSS 24 h ≤ 1) was able to identify 39% of the patients that achieved good functional outcome (sensitivity) at a specificity of 90% with resulting Youden index of 0.30 (95% CI 0.25–0.34.25.34), positive and negative likelihood (LR+ and LR-) were 4.02 and 0.68. dENI (NIHSS decrease of ≥10 or NIHSS 24 h = 0) achieved a sensitivity of 23% at a specificity of 95% with Youden index of 0.17 (0.14–0.21.14.21) and LR+/LR- of 4.22/0.82 to identify patients with good functional outcome.

### Excellent outcome ENI definitions

mENI was able to identify 46% of the patients that achieved excellent functional outcome (sensitivity) at a specificity of 87% with resulting Youden index of 0.33 (0.27 - 0.38) and LR+/LR- of 3.56/0.63.

dENI achieved a sensitivity of 26% at a specificity of 93% with Youden index of 0.19 (0.14 - 0.23) and LR+/LR- of 3.48/0.80 to identify patients with excellent functional outcome.

### Thresholds-based analysis for continuous NIHSS surrogates

Thresholds-based analysis for NIHSS at admission, NIHSS at 24 h, ΔNIHSS and NIHSS percentage change showed the following predictive performance: NIHSS at 24 h had the highest discriminatory power to identify patients with good long-term functional outcome at a threshold of NIHSS at 24 h ≤ 8. Sensitivity and specificity were 86% and 82%, respectively, with Youden index of 0.65 (0.58 - 0.73) and LR+/LR- of 2.88/0.20. Second highest discriminatory power was observed for NIHSS relative change with ROC AUC of 0.77 (0.74 - 0.79) and sensitivity, specificity and Youden index of 67%, 77% and 0.73 (0.70 - 0.75) at a threshold of a 30% decrease in baseline NIHSS to NIHSS at 24 h LR+/LR- were 2.88/0.43. Discriminatory power for excellent outcome (mRS 0–1 at 90 days) was equivalent to results reported for mRS 0–2 with slightly better results for the binary mENI/dENI definitions. The optimal cut-offs were different for NIHSS at 24 h with 7 and NIHSS percentage change with −33%.

### Influence of age and severity of initial neurological symptoms

**Fig. 3:**
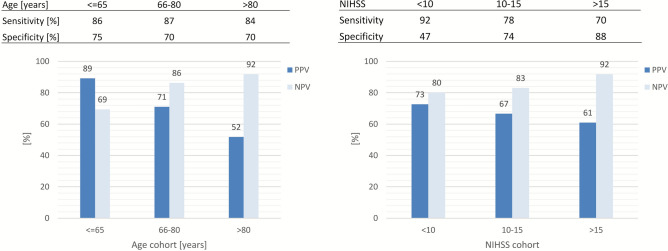
Discriminatory power of threshold NIHSS at 24 h ≤8 stratified by age (<=65y, 66y-80y and >80y) and stroke severity subgroups (NIHSS admission <10, 10–15 and >15). Bar charts show positive predictive value (PPV) and negative predictive value (NPV).

To evaluate the influence of age on discriminatory power of NIHSS at 24 h ≤8, predictive performance metrics were stratified by age and stroke severity subgroups (Fig. 3). For patients aged ≤65 years positive predictive value (PPV) was 89% and negative predictive value (NPV) was 69%, while specificity and sensitivity were 75% and 86%. With higher age, PPV decreased to 70% and NPV increased to 92% for patients older than 80 years while sensitivity and specificity remained comparatively stable.

If stratified by severity of initial symptoms, sensitivity decreased from 92% to 70%, while specificity increased for patients with more severe initial symptoms from 47% to 88%. PPV and NPV changed comparatively less with 73% and 80% for patients with NIHSS at admission <10 and 61% and 92%, respectively, for patients with NIHSS at admission >15.

### Discordance between 24 h NIHSS and 90-day outcomes

In the study population, N = 698 (55%) of the patients had an NIHSS ≤8 at 24 h, good functional outcome as suggested by surrogate NIHSS ≤8 at 24 h was achieved by N = 488 of these patients (70%). In total N = 640 (50%) of the patients had an NIHSS ≤7 at 24 h with N = 344 patients (53%) achieving excellent functional outcome at 90d. Vice versa, for N = 210 patients (30%) of patients with 24 h NIHSS ≤8 and N = 296 patients (46%) with 24 h NIHSS ≤7, the mRS at 90 d was >2 and >1, respectively, indicating discordance between early neurological status and functional outcome. Interestingly, 35% of patients with a 24 h NIHSS >10 and 22% of those with a 24 h NIHSS >15 still achieved functional independence at 90 days (mRS 0–2), despite presenting with severe neurological deficits at 24 hours.

Multivariable stepwise logistic regression was conducted to identify independent factors that are a) significantly associated with reaching the threshold of NIHSS ≤8 at 24 h, b) predictors for discordant functional outcome (90 days mRS >2) in patients with promising early neurological development (NIHSS at 24 h ≤8), c) significantly associated with reaching the threshold of NIHSS ≤7 at 24 h and d) predictors for discordant functionaloutcome (90 days mRS >1) in patients with promising early neurological development (NIHSS at 24 h ≤7).

**Fig. 4:**
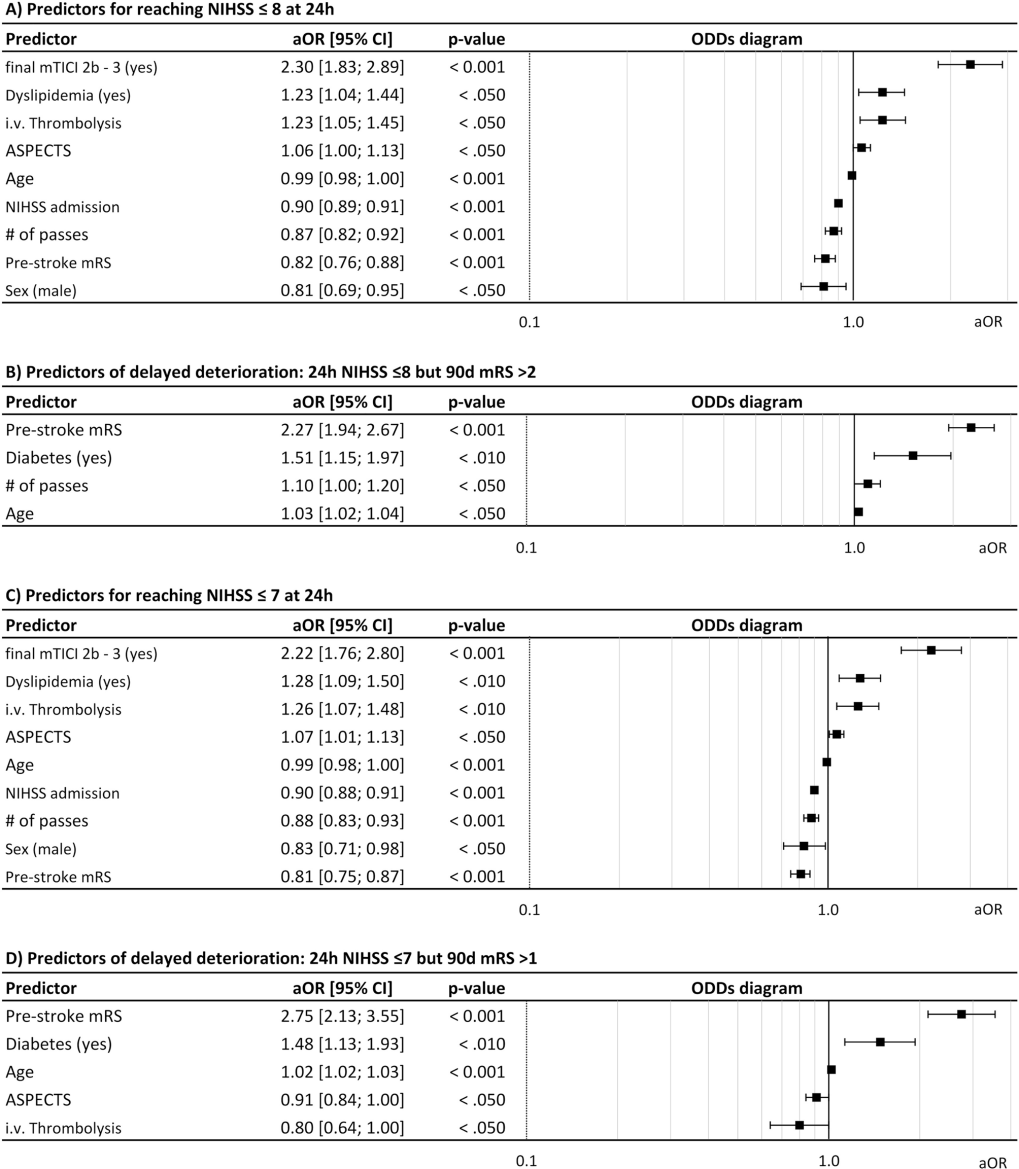
**A**–**D** Clinical predictors of reaching 24 h NIHSS thresholds ≤8 (Fig. 4 A) and ≤7 (Fig. 4 C) and of delayed/unexpected deterioration with 90 d mRS >2 (Fig. 4B) and >1 (Fig.4D), multivariable logistic regression.

Results showed that lower age (OR [95% CI] = 0.99 [0.98; 1]), lower pre-mRS (0.82 [0.76; 0.88]), lower NIHSS at admission (0.82 [0.76; 0.88] per NIHSS point), lower number of passes (0.87 [0.82; 0.92]), dyslipidemia (1.23 [1.04; 1.44]), i.v. thrombolysis (1.23 [1.05; 1.45]), higher ASPECTS (1.06 [1.00; 1.13]) and successful recanalization (2.30 [1.83; 2.80]) were significantly associated with higher probability of reaching NIHSS ≤8 at 24h. Male sex was associated with a lower probability of reaching NIHSS ≤8 at 24 h (0.81 [0.69; 0.95]) (Fig. 4A). The same predictors were identified for reaching NIHSS ≤7 at 24 h (Fig. 4C).

For patients achieving a promising early neurological status with NIHSS ≤8 at 24 h, higher age (1.03 [1.02; 1.04]), higher pre- stroke mRS (2.27 [1.94; 2.67]), higher number of passes (1.1 [1.00; 1.20]) and comorbidity diabetes mellitus (1.51 [1.15; 1.97]) increased risk for discordant functional outcome (mRS >2 at day 90) (Fig. 4B). For patients achieving a promising early neurological status with NIHSS ≤7 at 24 h, the same predictors with comparable odds ratios were identified for increasing the risk for long-term functional outcome with mRS >1 at day 90 (Fig. 4D). In addition, lower ASPECTS (0.91 [0.84; 1.00]) and no administration of i.v. thrombolysis (0.80 [0.64; 1.00]) were associated with discordant functional outcome.

## Discussion

Our analysis aimed to evaluate discriminatory power of early surrogates for predicting long-term functional outcome in patients with MCA-M2 occlusion based on a large multicenter cohort from the GSR registry. Compared to the HERMES MCA-M2 meta-analysis^4^, our cohort was older (74.3y vs. 66.0y), had a lower NIHSS at admission (10.7 vs. 14.4) and slightly higher ASPECTS (8.8 vs. 8.6). However, the rate of functional independence with mRS 0–2 at day 90 was lower with 45% vs. 58%. This observation might be explained by higher age as well as by more strict inclusion criteria of RCTs compared to registry data that reflect daily clinical practice. Three recent randomized controlled trials (DISTAL^12^, ESCAPE-MeVO^13^, DISCOUNT^14^) also included patients with MCA-M2 occlusions. In DISTAL, 71.6% of patients randomized to MT plus BMT had MCA-M2–M4 occlusions. Compared with our cohort, these patients were older (77.0 vs. 74.3 years) and presented with lower admission NIHSS scores (6.0 vs. 10.7), yet the rate of functional independence at 90 days was similar (57% vs. 58%). In ESCAPE-MeVO, patients randomized to MT plus BMT had MCA-M2 (50.2%) or M3 (35.6%) occlusions. Their age was comparable to ours (74.0 vs. 74.3 years), while admission NIHSS was lower (8.0 vs. 10.7). Functional independence rates were slightly lower than in our cohort (54% vs. 58%). Similar to presented results for anterior circulation stroke^15,19^ and posterior circulation stroke^17^, our results indicate that the NIHSS at 24 h outperforms other early clinical surrogates including binary and continuous definitions of ENI for predicting good (mRS 0–2) and excellent (mRS 0–1) outcome specifically in patients with MCA-M2 occlusion.

The prognostic value of continuous ENI definitions in our cohort was slightly lower, but comparable with published results in patients with LVO with the highest AUC for 24 h NIHSS of 0.85 in our cohort vs. AUC ranges of 0.86–0.89.86.89, followed by an AUC of 0.77 vs. 0.81–0.84.81.84 for NIHSS percentage change and an AUC of 0.71 vs. 0.73–0.77.73.77 for absolute NIHSS change^15,19,34^. This finding highlights the limitation of absolute NIHSS point changes as surrogate for functional outcome. Absolute NIHSS point changes reflect disproportionally the improvement in patients with severe strokes slightly reducing the NIHSS without significant impact on long-term functional outcome. Furthermore, the neurological status at admission does not reflect the therapy effect of thrombectomy and IVT and therefore might lead to a reduction in prognostic value of pre-therapeutic assessments in patients receiving MT^35,36^.

The observed optimal cut-off value to predict good functional outcome for MCA-M2 occlusion of 24 h NIHSS ≤8 is identical to the corresponding cut-off value published by recent studies for anterior circulation stroke with higher sensitivity in our MCA-M2 cohort (86.2% vs. 80.5–81.3.5.3%) but considerably lower specificity (70.1% vs. 80.0–82.0.0.0%)^15,19^. This finding highlights the strength of this cut-off as it seems to perform stable across subgroups.

A previous study by Wang et al.^37^ evaluating patients with distal vessel occlusion identified mENI as significant independent predictor of good and excellent functional outcome. However, they did not include any other ENI definitions and did not present any information in terms of predictive performance such as AUC, sensitivity, or specificity of mENI. This is a strong limitation because binary ENI definitions tend to have a high specificity but low sensitivity and should be rather considered a treatment effect proxy than a variable with predictive value. Furthermore, because baseline NIHSS was relatively low in this MCA-M2 cohort, only a small proportion of patients fulfilled the dENI and mENI criteria. These definitions were originally developed in populations with higher baseline stroke severity, where large absolute improvements in NIHSS are more likely to occur. In the context of moderate strokes, smaller but clinically meaningful improvements may not be captured by these thresholds, underscoring their limited applicability in cohorts such as ours.

In DMVO strokes the area that is affected by ischemia is smaller than in LVO strokes and therefore the expected clinical outcomes are possibly better^38^. In consequence, it has been proposed to consider a more restrictive outcome measure such as “excellent outcome”, i.e. mRS 0–1 in these patient cohorts^20^.

Accordingly, we also conducted corresponding analyses for prediction of excellent outcome defined by mRS 0–1 in this specific patient collective. Cut-offs for prediction of good and excellent outcomes differed by one NIHSS point in our patient cohort, while prognostic value in terms of AUC values were comparable for good and excellent functional outcome.

Predictors for reaching both, the identified cutoff of 24 h NIHSS ≤8 for good outcome and ≤7 for excellent outcome were lower age, lower pre-stroke mRS, lower baseline NIHSS, number of recanalization attempts, successful recanalization, dyslipidemia and i.v. thrombolysis. In octogenarians as well as severely affected patients (initial NIHSS scores ≥15), our results show a clear decrease in PPV and an increase in NPV, indicating higher rates of false positive and true negative calls in these subgroups. This observation might be attributed to dynamic NIHSS changes in these subgroups caused by multimorbidity, hospital-acquired complication and other adverse events related to severe strokes that reduce the probability of good functional outcome despite promising neurological status at 24h. Comparably stable sensitivities and specificities reflect the overall lower incidence of good functional outcome in older patients and patients with severe initial symptoms.

Furthermore, this study provides additional insight into why patients with MCA-M2 occlusion may experience a poor functional outcome despite achieving promising early neurological status at 24h. Functional outcome of mRS >2 despite achieving the 24h-NIHSS ≤ 8 surrogate was observed in about 30 % of our patients and in 46% of patients reaching the 24h-NIHSS ≤7 surrogate for excellent functional outcome, which is clearly higher than corresponding frequencies published by Meyer^15^ and Hendrix^19^ of 15.5% an 16%, respectively. This finding indicates that in patients with MCA-M2 occlusion, the post-acute treatment phase might be of similar importance as the acute treatment phase. It can be assumed that the NIHSS at 24 h cannot capture external effects and adverse events during rehabilitation^18,39^. Additionally, continuous cerebral hypoperfusion and subsequent infarction progress might cause delayed neurological deterioration beyond 24h^40^. These prolonged pathophysiological processes may therefore impair the prognostic value of 24 h NIHSS for long-term functional outcome.

Multivariable analysis was performed to evaluate predictors of a poor functional outcome despite achieving the NIHSS surrogate. According to our data, advanced age, diabetes mellitus and higher pre-mRS were identified as risk factors for reversing the prognosis from good to poor outcome (mRS 4–6) despite reaching the threshold of 8 NIHSS points at 24 h after MT. The same predictors were identified for a 90 d mRS of >1 despite reaching the threshold of 7 NIHSS points at 24h. This finding is in line with previous studies showing that probability of good outcome appears to decline with advancing age despite procedural success with complete reperfusion^15,19,41,42^. Furthermore, diabetes is associated with microvascular dysfunction. Higher rates of microangiopathy-related complications during treatment and post-interventional adverse events might be an explanation for higher risk of unfavorable outcome despite promising early neurological status. It has been shown that higher blood glucose levels are associated with worse clinical outcome^43^, but the pathophysiological pathways are not fully understood yet. Recent studies suggest that higher blood glucose levels trigger increased edema formation and might thereby lead to worse functional outcome^44,45^.

Our findings underscore the significance of conducting clinical assessments 24 h post MT, emphasizing that the NIHSS score at this timepoint may identify patients with promising prognoses, regardless of their baseline scores. This provides treating physicians with predictive insights for tailored patient management strategies. Furthermore, clinical assessment at 24 hours after MT may be valuable for clinical research. Consistent with our findings, both DISTAL and ESCAPE-MeVO incorporated the 24-h NIHSS as a secondary outcome measure. Future trial designs could similarly adopt the 24-h NIHSS as a secondary endpoint, using early neurological status as a surrogate for long-term outcome in datasets where 90-day follow-up is unavailable. Our study has limitations. First, all data were derived from a retrospective registry study design. Second, all clinical parameters including mRS and NIHSS were site reported parameters that might suffer from site related bias due to limited inter- and intra-rater reliability. Third, the exact anatomical definition of the MCA-M2 segment remains a matter of debate, and more detailed assessments, such as branch dominance, are not captured within the registry protocol. MCA-M2Due to numerous anatomical variants of the MCA, cohort definitions with exactly the same branching and vessel diameter parameters might not be possible in practice. Furthermore, results of the recent trials (DISTAL^12^, ESCAPE-MeVO^13^, DISCOUNT^14^) suggest that optimal patient selection for thrombectomy may not depend primarily on a more refined anatomical delineation of the MCA-M2 segment, but rather on additional parameters such as the degree of neurological impairment and the eloquence of the hypoperfused territory, which may better capture the clinical relevance of distal and medium vessel occlusions. Fourth, the potential hemispheric bias of the NIHSS should be acknowledged. Because the scale emphasizes motor and language functions, right-hemispheric deficits such as neglect and visuospatial impairment may be detected less reliably. This asymmetry may lead to underestimation of clinically relevant deficits in right-sided MCA-MCA-M2 occlusions and could, in principle, influence the prognostic precision of early NIHSS-based surrogate markers.

Although our left–right subgroup analysis demonstrated similar predictive performance across hemispheres, the conceptual limitation of the NIHSS remains relevant and should be considered when interpreting early neurological assessments in distal or eloquent-vessel occlusions.

Fifth, mRS assessment is heavily weighted toward motor functions, which might complicate comparisons of mRS and NIHSS. Sixth, only cases with availability of all required data points were included in the analysis. Exclusion of patients with missing data points (including lost to follow-up) might introduce bias to the reported results and reduce generalizability of findings. Seventh, our analysis was limited to patients treated with MT. In light of the recently reported DISTAL and ESCAPE-MeVO results, which did not demonstrate superiority of MT plus BMT over BMT alone, it would be of scientific interest to perform a similar analysis in patients managed with BMT only. Eighth, intubation status at 24 h was not available, which may affect comparability of NIHSS scores for patients intubated at 24h.

## Conclusion

Our study results showed that the NIHSS at 24 h with an optimal threshold of ≤8 provides the most reliable prognosis in predicting good long-term functional outcome for patients with MCA-M2 occlusion undergoing MT. Our data therefore propose the same metric and identical optimal NIHSS threshold value as reported for MCA large vessel occlusions. In addition, our data suggests an optimal threshold of ≤7 for predicting excellent long-term functional outcome for patients with MCA-M2 occlusion. Thus, similar to proximal MCA occlusions, the neurological assessment at 24 h post intervention is of high value for treating physicians to predict functional outcome in patients with MCA-M2 occlusions. Prognostic value of 24 h NIHSS was reduced by the following factors: advanced age, diabetes mellitus and higher pre-stroke mRS increased risk for discordant functional outcome after 24h.

## Supplementary Information

Supplementary Information.

## Data Availability

The data that support the findings of this study are available upon reasonable request from the corresponding author after approval of the GSR-ET registry.
