# Supplementary material for: Early surrogates of functional outcome after thrombectomy for MCA-M2 occlusions
Source: Sci Rep. 2026 Feb 17;16:6662. doi: 10.1038/s41598-025-34777-8 (PMC12914009; doi:10.1038/s41598-025-34777-8)
Supplement: Supplementary file 1 — Supplementary Information. [file 41598_2025_34777_MOESM1_ESM.pdf]

Supplementary Table S1: Comparison of clinical characteristics of included and excluded cohorts

	Included (N=1268)	Excluded (N=705)	Full cohort with M2 occlusion (N=1973)	p value
Age				0.665
- N-Miss	0	4	4	
- Mean (SD)	74.3 (12.7)	74.1 (12.8)	74.2 (12.7)	
- Q1, Q3	68.0, 83.0	67.0, 83.0	68.0, 83.0	
Sex (f)	651 (51%)	364 (52%)	1015 (52%)	0.828
- N-Miss	0	3	3	
NIHSS admission				0.376
- N-Miss	0	38	38	
- Mean (SD)	10.7 (6.1)	11.0 (6.4)	10.8 (6.2)	
- Q1, Q3	6.0, 15.0	6.0, 15.0	6.0, 15.0	
Comorbidity hypertension	993 (78%)	545 (80%)	1538 (79%)	0.285
- N-Miss	0	27	27	
Comorbidity diabetes	289 (23%)	175 (26%)	464 (24%)	0.072
- N-Miss	0	44	44	
Comorbidity dyslipidaemia	594 (47%)	289 (45%)	883 (46%)	0.366
- N-Miss	0	58	58	
Comorbidity atrial fibrillation	570 (45%)	292 (45%)	862 (45%)	0.876
- N-Miss	0	50	50	
ASPECTS				0.754
- N-Miss	0	145	145	
- Mean (SD)	8.8 (1.4)	8.8 (1.4)	8.8 (1.4)	
- Q1, Q3	8.0, 10.0	8.0, 10.0	8.0, 10.0	
Occluded vessel side right	534 (42%)	310 (44%)	844 (43%)	0.424
- N-Miss	0	0	0	
Intravenous Thrombolysis	609 (48%)	317 (45%)	926 (47%)	0.232
- N-Miss	0	4	4	
Number of passes				0.344
- N-Miss	0	130	130	
- Mean (SD)	2.0 (1.5)	1.9 (1.4)	1.9 (1.5)	
- Q1, Q3	1.0, 3.0	1.0, 2.0	1.0, 3.0	
mTICI 2b-3	1064 (84%)	553 (83%)	1617 (84%)	0.670
- N-Miss	0	40	40	
Treatment AE	257 (20%)	134 (19%)	391 (20%)	0.594
- N-Miss	0	5	5	
24h NIHSS				0.081
- N-Miss	0	160	160	
- Mean (SD)	10.0 (9.2)	9.2 (8.6)	9.8 (9.0)	
- Q1, Q3	3.0, 15.0	3.0, 14.0	3.0, 15.0	
90d mRS				0.174
- N-Miss	0	334	334	
- Mean (SD)	3.0 (2.1)	3.2 (2.3)	3.1 (2.2)	

- Q1, Q3	1.0, 5.0	1.0, 6.0	1.0, 5.0	
90d mRS 0-1	120 (32%)	387 (31%)	507 (31%)	0.504
- N-Miss	0	334	334	
90d mRS 0-2	160 (43%)	566 (45%)	726 (44%)	0.607
- N-Miss	0	334	334	
